# “It’s harder for the likes of us”: racially minoritised stem cell donation as ethico-racial imperative

**DOI:** 10.1057/s41292-021-00241-9

**Published:** 2021-07-13

**Authors:** Ros Williams

**Affiliations:** Department of Sociological Studies, The University of Sheffield, Elmfield Building, Northumberland Road, Sheffield, S10 2TU UK

**Keywords:** Donation, Bone marrow, Race, Stem cells, STS, Kinship, Relatedness

## Abstract

How best are we to understand appeals to participate in a biomedical project that are based both on invoking shared racial identity, and on framing engagement as the clear moral course of action? Stem cell donor recruitment, which often focuses on engaging racially minoritised communities, provides useful insight into this question. This article proposes that it is not an essential mutual racial identity between the person asking and the person asked at play. Rather, it is the creative ‘doing’ of relatedness between people at the scale of race as well as family that coalesces into powerful appeals to participate. Through analysis of ethnographic, documentary and social media data, the paper argues that this work relies at least partly on framing donation as a duty of being part of a racialised community, which I describe here as an *ethico-racial imperative*, in which both race and responsibility become intertwined to compel participation in the biomedical project of donor registration.

**Supplementary Information:**

The online version contains supplementary material available at 10.1057/s41292-021-00241-9.

## Introduction

How best are we to understand appeals to participate in a biomedical project that are based both on invoking shared racial identity, and on framing engagement as the clear moral course of action? Stem cell donor recruitment, which often focuses on engaging racially minoritised communities, provides useful insight into this question. This article proposes that it is not simply a mutual racial identity between the person asking and the person asked at play. Rather, it is the creative ‘doing’ of relatedness between people at the scale of race as well as family that coalesces into powerful appeals to participate.

Bone marrow or haematopoietic stem cell (herein, SC) transplantation is a well-established treatment for haematological malignancies like blood cancer.^1^ The treatment relies on the recipient receiving genetically-matched SCs, often taken from a donor from within the patient’s family, or via a global network of registries. In cases where clinicians resort to the registries, white patients are understood to be more likely to find a matching donor than racially minoritised patients, a situation framed in policy in different global north^2^ countries as a health injustice or “unmet need” (e.g. Smith 2018, see also Williams 2015).

This situation is considered to exist partly because there are proportionally fewer minoritised donors signed up to registries. As such, as in other places, there has been a push in UK civic society to register more donors of “ethnic minority” (common terminology in UK public and policy discourse to refer to people racialised as other than the majority ‘White British’ population), thereby increasing genetic diversity to the registries. This work, predominantly undertaken by individual campaigners and more established charities through face-to-face and social media recruitment activities, is the focus of this paper, which, like others in this special issue, offers an account of race being put to work *beyond* the lab.

The paper argues that efforts to engage minoritised donors rely at least in part on framing donation as a duty of being part of a racialised community. I describe this here as an *ethico-racial imperative* in which both race and responsibility become intertwined to compel action—in this case, participation in the biomedical project of SC donor registration. Importantly, this imperative is founded upon an expansive notion of relatedness grace of biomedical developments in which race endures (see Franklin and McKinnon 2001, Rabinow 1992, Nash 2005), and wherein participation is understood at least partly as an act of solidarity.

The paper begins by foregrounding how SC transplantation science is entangled with notions of relatedness and race, before explaining how racialised inequality is understood to have emerged with the establishment of SC registries since the 1970s. It then outlines the conceptual terrain upon which this paper is built, unpacking some of the literature that has grappled with how race and relatedness are mutually enacted in efforts to engage minoritised communities in various biomedical projects. The paper’s empirical section explores—via analysis of ethnographic, interview, documentary, and social media data—the efforts of different UK-based actors who mobilise this racialised framing that emerges from the lab, to encourage racially minoritised SC donor registration.

### Relatedness, race and stem cell registries

To understand race’s mobilisation beyond the lab in this context, it is first necessary to foreground how race became seen as a consequential element *within* the science of SC transplantation. Here, notions of relatedness within both families *and* racialised populations animate much of the understanding of tissue compatibility. The now well-established treatment protocol of SC transplantation emerges from early twentieth century concerns with the effect of nuclear material (Kraft 2009). Early experimentation used animal models (see Storb et al. 1971) and human trials (Thomas 1957) to inject healthy donor bone marrow cells into recipients with blood malignancies. Through this, researchers moved towards an understanding simultaneously emerging in the nascent discipline of immunology that two beings could have some gradient of ‘compatibility’ (Anderson and MacKay 2014), and that relatedness played a role: cells from related donors—in canine models, a dog from the same litter and in humans, a biological sibling—were thought most likely to be an adequate match (see Williams 2018).

The dominant model of immunological understanding explained this thus: donor/recipient compatibility is determined by their human leukocyte antigen (HLA) types. Using molecular analysis, an individual’s saliva or blood can be HLA-typed. The formation of protein sequences on cell surfaces—used to determine self from other—is guided by the HLA gene complex (see Howard, 2015). As per current consensus, individuals inherit maternal and paternal HLA alleles (Erlich 2012, p. 2), and their HLA type comprises these. Both recipient and donor are HLA-typed, and their compatibility measured by their HLA type similarity. Full biological siblings have a 0.25 chance of being a match, inheriting the same combination of alleles. Relatedness, then, is a key fulcrum around which the notion of genetic compatibility pivots.

HLA understanding developed in the twentieth century through immunologists’ efforts to “trace the global distribution of human polymorphism, or genome diversity” (Anderson and MacKay 2014, p. 129). This would culminate in tabulations of HLA distributions within different ‘populations’, a word invoked by researchers keen to distance themselves from words like race (e.g. Cavalli-Sforza et al. 1994). Nonetheless, these efforts would lay foundations for an understanding that if indeed two *un*related strangers have similar HLA types, they likely share membership of a specific ‘population’: “the likelihood of HLA matching is greatest with a donor from the patient’s racial and ethnic group” (Gragert et al. 2014., NB. the conflation of ‘racial’ *and* ‘ethnic’ here betrays the collision of these terms in the field, a point to which I return shortly). In effect, then, HLA mapping highlighted similarities within particular populations of nominally unrelated individuals, producing a modality of relatedness, implicit in the metaphors of population ‘trees’ and ‘roots’ calculated through ‘coefficients of kinship’ (Cavalli-Sforza et al. 1994), that relies up and reinvigorates the notion of race.

Alongside this, it became apparent that for SC transplantation to work at scale, an infrastructure through which to locate donors was needed (Pavlů et al. 2011). In the 1970s, the first SC registry was developed in the UK: Shirley Nolan, whose young child needed a transplant, established a newspaper campaign to recruit volunteers for the first database of potential unrelated donors. Though her work never rendered her son’s match, the effort would later be described as the ‘model’ for the many registries following it (Goldman 2002). Nolan’s work, driven by her relationship with her son, speaks to the work of non-scientific actors—operationalising knowledge produced within lab and clinical settings—in establishing an international network comprising around 40 million would-be donors across nearly 100 registries in 55 countries (World Marrow Donor Association 2021). Yet across the global network of registries, there is understood to be insufficient HLA diversity to ensure appropriately matched donors for all patients currently looking for a match (see Williams 2015).

In the UK, for example, racially minoritised patients are deemed less likely to find a match than their white counterparts because there are disproportionately fewer minoritised donors on the registries, leading to fewer possible matches for minoritised patients. UK policy (e.g. Smith 2018) describes this as an ‘unmet need’ borne of historical mistrust of health institutions amongst many racially minoritised people.^3^ Key actors emerging to address this need include the *African Caribbean Leukaemia Trust* (ACLT) and *Race Against Blood Cancer* (RABC), charities that work with registries. Alongside them, various (often racially minoritised) individuals requiring transplants but unable to find matches have established campaigns to locate donors, often engaging ‘traditional’ and social media in their efforts. This paper explores the UK context of this work of SC donor recruitment.

In the next section, I lay out the conceptual framing underpinning this analysis. First, however, it is useful to clarify this article’s use of the terms “race” and “ethnicity”, exploring as it does the UK context of this work where such words take on a specific meaning. Since the 1980s in the UK, the term BME (“Black and Minority Ethnic”) had been used both in policy and public discourse to describe individuals and groups who are racially minoritised (i.e. not of the majority ‘White British’ population). More recently this has been superseded by BAME (“Black, Asian and Minority Ethnic”), an acronym combining the racial and phenotypic ‘Black’, geographic ‘Asian’ as well as the term ‘ethnic’. The debate continues as to whether the simpler “ethnic minority” would be a more appropriate and inclusive term for those describing groups and researching racial inequity (see Aspinall 2021).

Sociologists of race and ethnicity note enduring disagreement amongst researchers about what is subsumed under these terms in the UK context (see Bloch and Solomos 2009, p. 4). Whilst ‘race’ is often a term associated with phenotypic and/or biological grouping and ethnicity is often associated with a *cultural* grouping, scholars have pointed to a tendency in public, academic and policy discourse in the UK, to use terms like ‘ethnicity’ to discuss fixed qualities in people without employing “disreputable notions of race” (Carter and Dyson 2011, p. 966). Similar insights have been made of the broader European context (see M’charek et al. 2014, p. 462), wherein race is seen as an *absent presence*, obfuscated by “the frequent coding of race discursively in other terms” such as those related to religion, nature and culture. Indeed, the terms race and ethnicity are used highly interchangeably in this field (see also Williams 2018), such that policy documents note that “HLA types are related to ethnicity” (UK Stem Cell Oversight Committee 2015, p. 75).

With this complexity in mind, the analytic portion of this paper predominantly uses the term ‘ethnic minority’ because this terminology is dominant amongst interlocutors in the field. This is done, building on interventions in the literature (Carter and Dyson 2011; M’charek et al. 2014), in the knowledge that the term may well be invoked by interlocutors and in data to gesture implicitly towards what Wade (2014, p. 592) describes as “idioms of nature”—shared, biologically transmissible qualities associated with ideas of race.

### Conceptualising an ethico-racial imperative

Relatedness and race are foregrounded in SC transplantation. The intersection of these themes in biomedical contexts, particularly their enrolment in entreaties to action, has also been the focus of interdisciplinary discussion within and between geography, anthropology, sociology and Science and Technology Studies (STS). Words like “kinship”, “relatedness” and “socialities” circulate in a literature concerned with how people bring understanding to, collectivise around, and act upon biomedical knowledge inflected with notions of race.

Suffusing many of these accounts is that a broadly conceived notion of ‘race’—and its mutuality between individuals—has currency through which appeals can be made. These appeals are performative, in the sense of encouraging particular actions, and in the sense of being the product of creative practices seeking to generate affect. These entreaties may also be informed by personal experience of suffering that have been causally linked back to one’s racial identity. They may also take on a normative tenor in which participation is framed as an ethical course of action. I suggest these appeals can be understood as an *ethico-racial imperative* in which calls to action and racial positioning—cast as biologically consequential—are interlaced and mutually reinforcing.

As biomedicine has expanded capacities for knowledge and action, ideas of relatedness and kin have been made evermore malleable (Franklin and McKinnon 2001) to accommodate, for example, new reproductive technologies (e.g. what makes somebody a “parent”?) and the expansion of genetic knowledge through ancestry testing (e.g. what constitutes an “ancestor”?). But relatedness and kinship have an enduring connection to our understandings of race, which precedes and now proliferates alongside these technological advances. As social anthropologist Peter Wade notes, this is because inherent within racial discourse are “ideas about inheritance and the family as a key medium of transmission” (2007, p. 8), a point acknowledged by Donna Haraway who locates kinship thinking within the discursive assemblage of race. Blood ties, for Haraway, are those “proteinacious threads extruded by the physical and historical passage of substance from one generation to the next, forming the great nested, organic collectives” (1994, p. 251). Relatedness and race are constitutive of one another as ideas. As such, as the bounds of relatedness expand, race remains central.

It should therefore be unsurprising that race is recognised as an organising principle in much literature exploring the relationship between relatedness, collectivity and health. Paul Rabinow’s notion of biosociality (1992) described how people were coalescing around knowledge produced by the then nascent genomics. People were united and propelled into action through genomic information and it was not, he thought, unthinkable that new socialities would gather around *this* allele or *that* chromosomal omission, often in a bid to end illness. New cultural classifications emerging from such technologies would crosscut, supersede, and redefine categories like race. Yet, as acknowledged since by other proponents of this line of argumentation, biosocialities need not be novel: “older categories of classification based [on] race or kinship inform, provide the framework for, exist in tension with new kinds of biological identities” (Gibbon and Novas 2008, p. 6). Race, and racialised communities, are made and remade through contemporary biological knowledge.

A rich seam of literature explores how this happens in various medical contexts. Social anthropologist Bob Simpson proposes, for example, that “when ideas of DNA, genomes, gene pools and populations cross over into popular ideas about culture and ethnicity” (2000, p. 6), ethnic identities might well become *imagined genetic communities*. This suggestion is important in outlining the work of imagination and invention in this context. In the case of SC transplantation, one will never know everybody who shares their HLA type, but through HLA maps and claims of different frequencies in different geographies one might be impelled—as I show below—towards acknowledging the wider, unknown group who may share one’s tissue type (and who may in the future need one’s help).

In the context of contemporary Brazil, for example, imagined genetic communities can articulate ties that transcend countries and states; a form of imagined *supranational* genetic community (Kent et al. 2014, p. 743) to be productively invoked in political arguments. Similarly, Melissa Creary’s analysis of Brazilian sickle cell disease highlights how colour and class, as well as biology, are mobilised by people living with sickle cell to make claims to the Brazilian state for health rights. Creary asserts that race is productive of a mode of genetic citizenship (see Heath et al. 2014) more aptly framed as “biocultural” citizenship. People mobilise in ways informed by their cultural positions and exigencies, and the intersection of health inequality and race provides a ripe formula for agitation: “a commonality is formed”, writes Creary, “only when we take into account the stratification of embodied suffering and the limits of existing political will to attend to that suffering” (2018, p. 130). Creary’s work speaks to the ambivalence inherent in the political mobilisation of racial identity as a means of achieving potentially liberatory ends.

Arguably, much of this literature seeks to trace the “practices through which different sorts of relatedness are enacted” (Nash 2005, p. 452). In this sense, kinship, far from essential, may be produced through creative practice (see Mason 2008; Carsten 2000). Tropes of substance like blood are still key in animating many (if not most) claims to kinship, but such relatedness “is created through both genealogical connection and cultural performance” (Wade 2005 p. 608) in which particular ways of feeling relatedness/kinship are performed, dramatic, choreographed to engender affect in an audience. Genetic ancestry testing exemplifies this when somebody’s results ‘prove’ (or ‘disprove’) their suspected ancestry and they record a YouTube video ‘revealing’ their ancestry “‘live’ on camera… with theatrical flair” (Nelson 2016, p. 100).

These performative underpinnings, which geographer Catherine Nash terms the “doing of kinship” (2005, p. 452), can have a normative orientation by linking identity to action (see Clarke et al. 2009, p. 23). As Nash notes, alongside disrupting/asserting claims to collective identity, these socialities generate “networks of obligation or affiliation” (2005, p. 457) wherein to act is to help address injustice. Asserting community in this way has an affective tonality which sociologist Thomas Lemke describes as the ‘semantic complex’ of guilt/duty (2013, p. 88). The inference of obligation, however, should give us pause to ask: to whom are entreaties made? As Lemke notes, those on the receiving end of information might feel compelled to deal with it “prudently and in accordance with moral imperatives” (2013, p. 88). Importantly, he highlights the asymmetry of such imperatives, which are likely to be felt more by certain groups than others.

This is particularly evident when biomedically asserted claims to relatedness are mobilised to prompt participation in specific projects, such as tissue donation or clinical trials. Participation in such projects constitute a set of “relations and modalities of the social” (Beck 2011, pp. 100–101, see also Simpson 2011) underwritten by similar claims to often heavily *racialised* biosociality, obligation and duty—what I describe here as an *ethico-racial imperative*; *ethical* in that a specific normatively framed course of action is emphasised, *racial* in that a specific set of individuals, focussed upon because of their racial identity, are the audience of the imperative.

An example of this can be found in how racially minoritised individuals are approached and enrolled into biomedical projects like clinical trials and tissue donation. Merz and Williams (2018), for example, argue that such efforts are often underwritten by claims that suffering within minoritised communities is directly exacerbated by a lack of these communities’ participation. This necessarily frames participation in biomedical projects as *the ethical decision*. From this starting point, as medical sociologist Jessie Cooper (2012) notes in her analysis of the “ethnic donor” in UK organ donation, minority “culture” (cast variously as ‘mistrust’ or ‘lack of awareness’) itself becomes a problem to be fixed through education and outreach, and the responsibility to address inequity a collective one for what are framed as “biologically, socially and culturally distinct and distinguishable communities” (Kierans and Cooper 2013, p. 14) —again gesturing towards the often corresponsive relationship of ‘race’ and ‘ethnicity’ in the UK context. When framed as a fixable problem, there is little space for legitimate refusal, which is instead cast as a form of ‘defection’ (Benjamin 2013) from ethically imperative participation, regardless of whether the grounds of refusal are ‘informed’, just as we might expect ‘consent’ to be (Benjamin 2016).

Importantly, however, the work to attract participation is often undertaken by people located within the communities of interest. STS scholar Steven Epstein’s (2008) work on ‘recruitmentology’ similarly explores the training of trial participants to recruit further participants from within their communities. Epstein describes one instance wherein *embajadoras* (ambassadors), Latina study participants, were trained to recruit fellow Latinas to the study. In line with Kierans and Cooper (2013), Epstein argues this constitutes a particular social episteme wherein that which becomes not simply knowable, but *recruitable*, is not the individual, but the social group to which they belong. The group becomes “a social actor, complete with interests, chosen representatives, and its own collective memory—an agent that must be engaged with and taken seriously” (2008, p. 812).

In the cases above, attempts to engage minoritised communities rest upon moral appeals that generate a sense of solidarity within a community of suffering, generally in relation to ongoing or potential health inequality (e.g. too few minoritised participants in pharmaceutical trials potentially leading to poorer outcomes for future minoritised users of new drugs). In this sense it is not necessarily just shared identity, but shared suffering, which might lead to framing engagement as a kind of imperative. As sociologist Ruha Benjamin reflects, “all kinship, in the end, is imaginary. Not faux, false, or inferior, but… a creative process of fashioning care and reciprocity” (2018, p. 64). It is, then, such entreaties to action (in this case, to registering as an SC donor) based on mutual racial identity and suffering—an *ethico-racial imperative*—that are the focus of the rest of this paper.

## Methods

The following is based on analysis of grey literature including 23 documents and reports produced by UK-based policymakers and SC registries (see supplementary material, Table S1), qualitative interviews with 17 actors in the field, and participant observation at donor drives^4^ in various locations in the UK with both the *African Caribbean Leukaemia Trust* and *Race Against Blood Cancer*, two UK-based charities working to increase SC registration amongst the UK’s racially minoritised population.

Additionally, arguments developed here build on analysis of three social media campaigns for individuals seeking matches. This includes a set of Tweets and Facebook posts for these campaigns, as well as a range of media coverage and blogs written on campaign websites (see supplementary material, Table S2).

Data were analysed in Nvivo using an abductive approach in which analysis was understood as an ‘inferential process’ informed by the analyst’s familiarity with literature in this empirical area (Timmermans and Tavory 2012). Ethical approval for this project was obtained from the author’s institution. Where individuals’ names have been used or social media campaigns have been identified, permission has been obtained from them to do so.

### Donor registration as an ethico-racial imperative

In the context of UK SC donor recruitment, non-scientific actors of ethnic minority background are framed as particularly effective interlocutors in generating engagement of ethnic minority people. The latest parliamentary review on the topic, *Ending the Silent Crisis* (Smith 2018) emphasises the role not of scientific, clinical, or statutory actors in trying to reach ethnic minority communities, but organisations working *within* these communities:

The rate of donor recruitment by BAME [Black, Asian and Minority Ethnic] grassroots and community organisations is high[…] Grassroots and community organisations’ knowledge of respective target audiences, and their dissociation from governmental institutions, generate higher levels of trust from BAME groups. This allows them to reach under-represented communities more effectively[…]. The message or the framing might be accurate but its effectiveness is dependent on the messenger and mode of delivery. Due to their integration in local communities, BAME grassroots and community organisations are best placed to judge the types of approach that will work best for which topics (Smith 2018, pp. 10–11)

Community organisations are framed in the report as valuable for their perceived capacity to generate ‘higher levels of trust’ due to their ‘dissociation’ from government. That ‘BAME grassroots and community organisations’ are ‘best placed’ in communicating with ‘target audiences’ infers that these organisations’ value also hinges on their members’ racial positioning. It is less the message that is seen to be at issue, than the messenger. This is emphasised by the *African Caribbean Leukaemia Trust* (ACLT), whose submitted comments featured in the report. They noted that

…external non-ethnic parties in working within Black communities has not resulted in significant increases in consenting donors. This is because of [statutory bodies] not understanding and appreciating these communities in relation to their history, culture, demographics, social nuances, etc. (Smith 2018: 9)

As with the *embajadoras* (Epstein 2008) whose position within clinical trial researchers’ communities of interest conferred a legitimacy that white researchers did not enjoy, those *not* integrated (i.e. not within the community, or part of ‘BAME organisations’) are not well-positioned to grasp the specificities of communities. In the case of SC recruitment, these specificities are framed as cultural (e.g. being able to make sense of history and ‘social nuances’), yet the work relies on recognising a biological quality (a preponderance of certain HLA types) in the targeted community, which minority-led organisations are seen as vital to latch into.

This is borne out in the interview account of an individual working at a SC registry, who collaborates with recruitment charities. They specifically articulate the superior recruitment capacity of ‘community groups’ over organisations:

[Our registry is] not necessarily seen as a […] trusted voice […] Our recruitment […] online, broadly reflects the UK populations, as we do around 13-14 per cent of minority ethnic backgrounds, so it just reflects the population because we aren’t able to be as targeted as we are with events where[…] we work with community groups,[…], and we have much more success with events with that.

This participant’s account describes the registry’s broader recruitment work, much of which is now done through internet advertising campaigns and the ordering of swabs online by registrants, leading to a majority White British recruitment (see Williams forthcoming). They express an inability to ‘target’ ethnic minority groups—they aren’t a ‘trusted voice’—so attempt to achieve their goal of increased ethnic minority recruitment by relying specifically on smaller recruitment charities who have ‘more success’ at physical events. Another registry worker makes a similar point, situating this in a broader issue of health engagement:

engaging minority ethnic communities in health-related things, whether that’s blood donation or whatever else, a lot of that is about the trust that different communities might have in either the NHS [National Health Service^5^] and health systems or in charities like [SC registries]… trusted groups like [community organisations] help us connect with those communities.

Here, recruitment charities are the ‘trusted groups’ that bridge institutions with individuals who might not otherwise trust them. These accounts surface how the capacity to make successful appeals can hinge both on community groups being from *within* ethnic minority communities, but also *without* the institutional setting of the state or larger charity sector. In this sense, not simply being *beyond the lab* but appearing external to the entire statutory apparatus is itself a central element of this work.

This also surfaces during donor drives. Take, for example, a *Race Against Blood Cancer* (RABC) donor drive I attend with a charity recruitment co-ordinator, a Black man in his thirties who helped build RABC after his close friend struggled to find a match. He has organised this drive at a multi-national bank headquarters in the City of London. Most of his drives are in corporate settings because he could have his local contact email employees to advise them of his presence (he explained that people might be “looking for an excuse to get away from their desks”).

This day, we are stationed halfway up this huge glass building in its cafeteria. Arriving before 10am, we are given a long table draped with a black sheet to lay out materials: forms for new registrants to complete, buccal swabs with which registrants provide saliva samples for HLA typing, and individual swab envelopes with donor ID barcodes that, once filled, will be sent to the registry for processing. The table is flanked on either side by the charity’s roller banners. They are red and black to match the charity’s branding. On one of them is a silhouetted figure. It is a feminine figure, hands on hips, hair tied up on her head so the outline of tight curls contrasts against the red. I read her as Black. The banner reads “JOIN THE STEM CELL DONOR REGISTER HERE”, with no mention or logo of the NHS or of the larger registries to which registrants’ data and saliva samples eventually flow.

We sit behind this table for five hours as the co-ordinator tries to get people’s attention to come over and register as donors with refrains like “Have you heard of us before”. If somebody answers his call (mostly, people ignore us), this leads to a brief introduction to the charity and the process of recruitment. It is notable that the co-ordinator invokes his organisation’s independent brand, both in his introductory pitch and decorative banners, in such a way that suggests, as the policy explicates, a value in the institutional distance between these organisations and the state.

This event, however, is also useful for thinking about how racial positionality is mobilised in recruitment. During the drive,

two young Black women were walking past, and [he] managed to catch their attention[…]he asked if they’d heard of the charity[…] They were obviously compelled by him and/or his message, which he quickly delivered, explaining the need for stem cell donors. One of the two women took a pen almost immediately, and started writing her details into the form[…] The undecided woman came back later on in the day – the only person who did return after saying they would. “Told you I’d come back!” she said to him. “I could tell you didn’t believe me!”. […Later,] I asked him whether he thought the two Black women who’d come over were more inclined to register because he’s Black. “100%. It’s a different cultural appeal - I appeal to ethnic minorities”. (fieldnotes, January 2020)

His assuredness that his racial positionality increased their inclination to register is summed up in the idea of being able to ‘appeal to ethnic minorities’ in a manner he presumably wouldn’t be able to do if he were white. Throughout the day, he draws on statistics fluently (this is a field saturated with statistics, see Williams 2016), telling the two women above about the limited odds for ethnic minority people seeking a match because there were fewer minority donors registered, and explaining minority patients are more likely to find their match with minority donors, thus infusing his appeal with the racialised understanding dominant in SC transplantation science.

Importantly, however, this work doesn’t always lead to successful engagement. Whilst the co-ordinator’s request generated action in the earlier extract, there was no guarantee that it always worked:

Not that long after we’d been there, a Black lady came over after [he] had asked if she’d heard of RABC […he] then tried to see if she’d register – he told her that it was “really important that we get Black people signing up”. “Minority ethnicity people are more likely not to find a match”. He reeled off some statistics – white people have a 1-in-1,000 chance of finding a match, but minority ethnicity people have a 1-in-200,000 chance of finding a match. Would she consider signing up? She said she was busy right now and had to go, but “you’ve made me feel guilty” so she’d tell her office mates. As she left, [he] said “she felt guilty, but not guilty enough to register!” (fieldnotes, January 2020)

Highlighting suffering through statistics did not work here as intended. Though it obviously did something—she claims to feel guilty—she is ‘not guilty enough’. Nonetheless, even though appeals may not always be successful, those working in the area (as we see in the interview abstracts with registry workers) consider recruitment appeals more effective than the (white) alternative. As the policy document above these fieldnote extracts highlight: to be part of the ethnic minority community makes somebody ‘best placed’ (or at least better placed than a ‘non-ethnic’ party) to make requests of that community (Smith 2018, p. 11).

As such, this work can be seen to entangle both the given recruiter’s externality to the institutions that require the donors (the NHS and registries) and their position *within* the racialised community of interest. Simultaneously, this hinges on an understanding of the innate biological value of having such people registered as donors, to increase the matching odds for other patients. The emphasis on the importance of a potential registrant’s decision—e.g. ‘really important that we get Black people signing up’—is also suggestive not only of the racial, but *ethical* tenor of these appeals. It is this that moves an appeal from simply a request, to an imperative. At the bank drive, the appeal is urgent: the table behind which we are sat is not just an information stand. It is a place where you can be swabbed and registered today, within minutes. A familiar refrain at these events is always that it’s better to get the swabs done today—the sooner done, the sooner the donor is searchable on the system for patients in need.

This sense of urgency, and its entanglement with race, permeates much individual campaigning too. Whilst this is often framed in terms of an individual’s prognosis (campaigners interviewed often described having only months before a SC transplant was no longer an option), need was also often framed in explicitly racial terms. For example, across various campaigns, celebrities of the same racial background as the would-be recipient are often approached (with limited success) to record such appeals. In an instance where celebrity attention *was* captured, British Asian actor Dev Patel (of *Lion* and *Slumdog Millionaire* fame) records a video (HelpVeerNow 2020) wherein he asks viewers to consider registering SC donors to help the young boy at the centre of the HelpVeerNow campaign who has not found a match.

A gentle plucked guitar soundtrack overlays this video, with the campaign’s website and social media handles presented neatly on the screen. Patel moves in and out of shot as text appears on the screen. He explains he is here to talk about “a very special young friend of [his] called Veer”. A picture of Veer appears on the screen, along with details of his diagnosis, age and location. The screen cycles to further text: “South Asians like Veer have only a 20% chance of finding a matching stem cell donor” before Patel appears, sat on a sofa and looking directly at the screen. His voice, earnest, slow and sober, explains “there are hardly any donors of Asian origin in the UK, and that's why I'm talking to all you guys today. We need to raise awareness within our communities and around the world”.

The video, shared on the HelpVeerNow Facebook page, attracts over 16,000 views. A mutual identity is mobilised here—between both the individual in need (Veer), the asker (Patel), and the asked (“South Asians”). It is demonstrative of how race is framed as a collective (‘our communities’), how engaging with SC registration is framed as a ‘need’, but also of the “doing” of kinship (Nash 2005) embodied in the video’s composition. Veer’s personal story is told, the seriousness of his situation echoed in the actor’s solemn delivery.

This sense of racialised obligation infuses much of the work of recruitment. As I have noted elsewhere (Merz and Williams 2018), this work is highly affective—trying to draw an audience into a narrative on highly personal and emotive terms often through creative practices. We see this in the recruitment co-ordinator’s exasperation with the woman who declined, insufficiently influenced by the ‘semantic complex’ of guilt/duty (Lemke 2013) in the co-ordinator’s words. Importantly though, much of this work is animated by more than mutual racial identity. It is, crucially, personal biography (often through bereavement or concern for a loved one currently seeking an SC donor) that often underwrites these appeals.

### Mobilising relatedness in recruitment

The campaigns and organisations discussed here emerge around specific patients requiring a SC donor, who place their inability to find one on their race. As such, the work undertaken must be understood as a response to a highly personal, familial issue refracted through the explanatory lens of race. In the case of Match4Lara, a campaign to locate a match for a young woman called Lara, much of the publicity was driven by and featured her family. In one Tweet sent out by a SC registry, people are invited to

Watch Lara's mum explain why we're urging those with a mixed ethnicity in particular to register #Match4Lara (DKMS UK 2016)

The Tweet links to a YouTube video of a donor drive run by the campaign (Match4Lara 2016). As with the video for the HelpVeerNow campaign, a gentle guitar track fills the audio between words in this video. Cut scenes move between close-ups of Match4Lara-branded t-shirts (warn by volunteers) and rubber charity wristbands (sold at drives to raise funds for the registries). The video pans tables populated with the same ephemera of swabs and forms described in the bank donor drive fieldnotes, above. A range of people in terms not only of race, but age and gender, swab their cheeks at the drive. Lara’s mother comes into shot, and explains that they expect to find Lara’s donor in the pool of ‘minority and mixed-race’ donors:

Hi, I’m Lara’s mother and we’re here today to get people onto the donor registry. Lara, my daughter, has leukaemia and she needs a stem cell donor urgently. Doctors have advised that the match is likely to be found in minority and mixed-race communities. It’s really easy and safe to register. You could save a life, and that’s why we’re here today. Please join a local drive or sign-up online.

It is notable that the appeal mobilises an apparently stable category that might be targeted (‘minority and mixed-race communities’) even though the notion of ‘mixed’ is itself inherently unstable (see Williams 2018) and arguably more diffuse than the community of interest to, say, the HelpVeerNow campaign. It serves to exemplify how, across these examples, a racialised subject is enacted, so that an appeal can be made to them. Incidentally, both the tweet and the video to which it links neatly demonstrate the interchangeability of “race” and “ethnicity” in the field, as the two sources uses the separate terms to gesture to the same audience and cause. The mother’s account also demonstrates how clinical knowledge (from ‘doctors’) regarding the importance of race in matching, filters through into this civic work.

The appeal, importantly, is also made by a parent with a child in need (also see Creary, this issue). This intertwining of race and family is central across much of this work. ACLT enrols the memory of the son of founders Orin Lewis and Beverley De-Gale who initially began campaigning to locate his match. Their child, Daniel, benefited from nine years of extended life after receiving his match. Pictures and videos of the organisers’ late son illustrate presentations, the banners placed behind the stands at events, and the take-home literature dotted neatly about tables at their drives. A slide in the organisation’s presentation is entitled with their son’s name (they tell me that speeches are integral to conveying their message. Indeed, they tend not to run drives if there is no opportunity for stage time—speaking to the value of the staged engagement in their work). It lists his diagnosis, his treatment pathway, and the date ACLT was launched. The slide features an image of him, as well as one of him and his mother, smiling and hugging.

The affection with which they talk of their child in our informal conversations, and his presence in their campaigning, speaks to the centrality of that relationship to their work. This is particularly evident at a drive at a Pentecostal church with a predominantly Black African congregation who fill around half the seats in a large hall that accommodates perhaps a thousand people. The congregation, along with myself and the charity volunteers I am sat with, have listened to the pastor speak for half an hour about a passage from Hebrews in the New Testament. He then introduces ACLT, reminding the congregation that “it is our duty” to register as blood, organ and SC donors. He calls up Orin, who joins the pastor on stage.

Orin receives a round of applause, and thanks the congregation. This is not the first time I have seen him in front of an audience, and he seems unfazed, speaking steadily and confidently about the purpose of ACLT’s visit. He asks for the audience to listen to somebody else before he gives further detail about the process of signing up, which the charity will be helping people to do today. Then he invites to the stage the bereaved father of another child, whom he introduces and asks to share his story. I make some notes of the bereaved father’s oratory, which I watch from the audience:

He explain[ed] how whilst his son was in [hospital], unable to find a match, there was a Black woman there, whose five month old child was suffering in the same way. And another Black lady with an older child, again unable to find a match. “It’s harder for the likes of us”, he said. The reason there were so few donors from Black backgrounds, he’d been told by somebody during his son’s treatment, was “because there are not many of us. But look at how many of us there are” he said, gesturing out across this church hall, filled with Africans and African-descent Londoners… I’ve watched how kids die… I didn’t [just] stand up for my son, I stood up for all our children. Children are our future, and it’s an investment in our future. (Donor drive, London. December 2019)

The father’s testimony—more apprehensive than Orin’s, suggesting the rawness of recent loss and less familiarity with the stage—recalls his lost child. It is more personal than the statistics that the co-ordinator used at the drive in the bank headquarters. The father’s account amplifies the nonsensical nature of his son’s inability to find a match (‘look at how many of us there are’), latching onto a “broader repertoire of invoking the slain to vivify collective action” (Benjamin 2018, p. 46). It also frames participation as an investment to ensure a common future, enacted by collective pronouns and physical gesture. It marries with the performances of the pastor, and Orin, and channels towards the ethical action of investment through participation.

This interpolation of race, family, shared suffering and duty speaks to the entanglement of race and relatedness in these appeals. Here, ‘degrees of relatedness’ surface as the words move between scales of family and race (Nash 2005). The narrative explicitly incorporates race— ‘a Black woman’—and familial relation—her child. It highlights the commonality of that identity and the attendant inequality in treatment access— ‘it’s harder for the likes of us’ to find a match—as well as emphasising that it was not just *his* son for whom he was speaking, but ‘for all our children’. The imperative laid out by this father takes place on a register of race as a degree of relatedness, to the extent that shared racial identity, insofar as these actions bring it into being, cannot be extricated from the tropes of family like parenthood that are used to reinforce it.

The value in drawing together these personal stories of suffering with race is evident when Orin returns to the microphone to thank his guest and continue presenting. It is a wide-ranging presentation wherein he describes the process of donation and shares pictures of people who did (and did not) find matches. He tells the story of when Daniel found his match from an unrelated donor, sharing happy images of their eventual meeting after his transplant. The transplant would extend his life several years. The inclusion of these narratives of ‘success’ suggest that suffering needn’t be the only theme of this work, though he concludes with a video that tells the fictional story of a young Black man in poor health. The actor’s white friend finds a match, whilst he lies is hospital. Struggling to breath, the actor asks the watcher “could you be my match?”, as mournful strings and piano play over his coughing (Could You Be My Match 2015). At the end of the video, Orin finishes with the following appeal:

“Be a positive role model to your community,” he said, looking out across the church hall, speaking slowly as if to ensure the message sank in. “Be a positive role model to your race”. He pointed out that[…] from today, who knew what might happen – one of the people who sign up later to be a stem cell donor could be a lifesaver[...] Beginning to draw the presentation to a close, he very soberly stated: “Time is running out for people, especially our people” (fieldnotes, December 2019)

He speaks slowly as he treads the stage and makes eye contact with audience members (*a la* Nelson’s (2016) “theatrical flair”). I locate in his words, which I hurriedly jot down, an effort to create at once both race and relatedness. This capacity to make such a weighty appeal is drawn here at least in part from Orin’s Blackness. Like with Dev Patel’s appeal to ‘our community’ of Asians, a shared identity between audience and speaker seems to be assumed: he can talk to his audience with collective nouns— ‘our people’—that somebody without his skin colour could not. The appeal is also founded upon narratives of family, bereavement, and suffering (see Creary 2018; Nelson 2011) but also orbits around an act (donor registration) that stands to mitigate this. I ask Orin about his approach during an interview. He explains:

I never repeat verbatim word-for-word whatever I’m going to say on stage, but […it] relates to a personal journey. Obviously via Daniel[…] trying to make people who are listening feel part of the potential of[…] giving the gift of life[…]. So I try to impart that feeling of not only what we went through, the darkest moments, but the uplifting possibility of what we experienced when Daniel’s donor was found and we met them[…]. So you try to bring your audience with you to experience just a little bit of your pain, the pain and glory, two extremes, and[…] create the vision for people to understand how low you can be, but the possibilities of how great it can be,[…] and the people feel “I could[…] be part of that”, either individually or as a collective.

There is, then, an explicit effort to ‘impart’ a feeling in these events and to draw attention not only to the ‘pain’, but to its inverse: the ‘glory’ of finding a match, to draw people to the act of registration that is framed as enabling it. It is highly *affective* work, then. Indeed, the speeches give me goosebumps, make my eyes wet. I am moved by the father’s story about his son; the sad video of the fictional, hospitalised young man; the happy family photographs of Daniel meeting his donor. It is also, apparently, highly *effective*: afterwards, we wait behind a long desk in the foyer with our ACLT t-shirts on, poised for the congregation to emerge once the pastor closes the service. Many visit the stall to complete a registration form and swab their cheeks. The drive registers 67 people that day from the larger present congregation of perhaps 500 people. It is a figure that seems to pleasantly surprise the volunteers as we count registration forms after the drive.

## Discussion and conclusion

The appeals considered here are explicit efforts to generate not only a mutual racial affiliation, but also a sense of responsibility. This happens across different examples of appeals to people of ostensibly different racial backgrounds. This paper has centralised some of the creative elements, from statistics to family photographs, the use of video, and real and fictional narratives, that are combined in this generative pursuit. But this is not simply to demonstrate how race, to follow Catherine Nash (2005), can be framed as a *degree* of relatedness, not entirely unlike family. It is also highly normative work, in that it explicitly encourages a specific pathway of action. In this way, the paper builds upon a range of scholarship from sociology, anthropology, geography, and STS to exemplify how appeals to engage in biomedical projects can be made on the grounds of both race and moral action, which I have here termed an *ethico-racial imperative*.

SC donor recruitment though, is more than just an example of an imperative in action. It operates as a key site where racialised inequity and efforts to address it are actively being played out. The work I have explored here is often laced with a concern to end or alleviate the evidently racialised suffering that compelled these individuals into this work in the first place. Think of the mother recording a YouTube video asking people to help her daughter, or the father recounting a story of his late son in a hospital bed. The drama of the stage, the stoking of affect, emerges both from bereavement and a desire to ‘invest’ (to borrow the bereaved father’s words above) in a shared, alternative future. The emphasis on suffering and the actions that might alleviate it is a key mode of this work. But it is important to recognise—as do Benjamin (2018) and others (see Clarke and Haraway 2018)—that this imperative is not simply a command to action, but an example of what Haraway terms “innovating enduring kin” (2015, p. 164). Shared identity is enacted through this work.

Perhaps a particular appeal works (think of the people who register after being asked to during a speech at a registration drive), or perhaps it does not (consider the woman who didn’t ‘feel guilty enough’ to register). Indeed, what I have not had opportunity to do in this intervention, but which clearly emerges as a necessary future focus from this paper’s argument, is explore why people might say “no” to appeals. This, I suspect, would require us to take seriously both the historical and contemporary contexts (see Fitzgerald et al. 2020; Benjamin 2016) in which minoritised individuals elect *not* to engage with biomedical projects like clinical trials, tissue donation and so on. Indeed, rationales for refusal are hinted towards in allusions across the above data to the notion that the state and institutions like the NHS and registries inspire less ‘trust’ than do community organisations.

At a moment where racial injustice and health are once again at the forefront of public discourse with the confluence of coronavirus and Black Lives Matter, it is worth asking what different kinds of action such imperatives are being put to work in service of. For example, as minoritised people are under-represented in clinical trials for coronavirus vaccines, whilst simultaneously being disproportionately hospitalised by the same disease, the narrative of racialised responsibility to engage as participants in trial research is stark in media discourse.^6^ Future work might also consider the nuances of how an ethico-racial imperative is deployed in the context of differently racialised groups, as well as in different national contexts and to engage people in different biomedical projects.

Perhaps the more immediate issue, then, is not the format or efficacy of the imperative, but understanding what brings it into being in the first place, and the assumptions upon which it rests. I began this paper by highlighting that SC transplantation science is highly racialised. It is infused with particular ways of knowing the social world and how it is organised (families, races). The science itself engages in a kind of metonymic alignment of genes and race that generates a sense of race as biologically essential. We can see this in the way the field frames compatibility: people are believed most likely to find their match within their own racial group (see Williams 2018, see also Hacking 2005). This racialised understanding comes to permeate recruitment work which, though taking place beyond the lab, carries and perpetuates this understanding. Here, I have been interested in critically interrogating the practices through which actors in this field generate collectivity with other minoritised individuals, and attempt—through recourse to a variety of resources like their own racialised identities and personal biographies—to engage these persons in SC donor registration, an act framed not simply as temporally urgent but as a moral duty befalling minoritised people in service of those with whom they are cast as genetically and racially alike.

It is widely accepted that access to SC transplant treatment is limited, and that this limitation plays out along racial lines. Ameliorating this lack of access is framed in UK policy as a highly specialised job. It is seen not a task for scientists, clinicians or the state, but for those of ethnic minority who mobilise the racialised framing of the issue emerging from the lab, and then operate *beyond* it (see the editorial, this issue). These individuals, it is thought, are more effectively placed to enter conversation with ‘their’ communities. I have shown here how such efforts can operate in practice.

The work of articulating this imperative, though, is generally undertaken by individuals who express having no alternative other than to act in this way, as there is nobody else there to do this work on their behalf. The state itself has effectively outsourced this labour to people thought better placed to do it: minoritised citizens who are now faced with the task of leading the charge against a racialised inequity in healthcare provision. These individuals draw upon the resources available (stories, identity, statistics) because there was no alternative for them. The work is central to memorialising a lost loved one in whose name others might find their matches, or it is the means through which a loved one’s life—imperilled by disease—could yet be saved. Ultimately, though any one of us may choose (not to) register as a donor, it is the very absence of choice that underwrites these calls to action. In this instance, it is as much an imperative for the person making the appeal as the person in receipt of it.

In close, then, it is apt to raise an uncomfortable and not easily answerable question. What does it tell us that so much of the ongoing and difficult work to ameliorate health inequalities is actively placed in the hands of racialised communities themselves, rather than framed as a collective onus borne by us all, regardless of how we identify or are read, to address the historical striations of inequity that our health systems so urgently need addressed?

## Supplementary Information

Below is the link to the electronic supplementary material.

Supplementary file1 (DOCX 19 kb)

## Footnotes

1 SCs are also variously referred to as blood stem cells, or bone marrow stem cells. SC treatment incorporates allogeneic transplants (where a recipient receives cells from a donor who may be related or unrelated), and autologous transplants (where the recipient’s own cells are transplanted back into them). This paper is specifically concerned with locating donors for so-called ‘unrelated’ transplants. See Passweg et al. (2018) for the most recent published treatment data in the European context.

2 The WHO acknowledges, however, that accessibility to treatment is not universal (World Health Organisation, n.d.). This inaccess can be viewed from two fronts: people can only access such a treatment in countries where SC transplant is an available therapy. There is also a preponderance of SC registries in the global north (see Petersdorf 2010), which compounds the issue of representation discussed in this paper.

3 There isn’t scope here for detailed discussion of the issue of mistrust in the context of tissue donation, but in the contemporary UK context, “hostile environment” policies in healthcare settings alongside data sharing agreements between health institutions and Government (see Fitzgerald et al. 2020) arguably compound more enduring concerns amongst some racially minoritised groups that the health system is not trustworthy.

4 Interviews with registry workers, recruitment charity workers, and those involved in individual patient campaigns, have been undertaken since September 2019 as part of a Wellcome Trust research fellowship. Initial ethnography garnered fieldnotes from participant observation at ACLT and RABC events since September 2019 in London, the Midlands or that have been streamed on their social media sites. The face-to-face component of fieldwork was suspended in March 2020 because of the coronavirus outbreak, which paused much SC recruitment activity globally (see Williams forthcoming).

5 See Fitzgerald et al. (2020) on the contemporary cultural politics of the NHS, particularly the ‘racial faultline’ of healthcare access.

6 Alongside coverage in national UK press that emphasises an underrepresentation of minoritised volunteers in clinical trials for coronavirus vaccines which draws in narratives of minoritised politicians appealing to ethnic minority publics to engage (e.g. Siddique 2020), ‘calls to action’ have been made by celebrities. One example is a video featuring British-Iranian comedian Omid Djalili and African American actor Whoopi Golberg who encourage Black and Asian audiences listening to register for research into the effects of coronavirus (see Centre for BME Health 2020).
