# Supplementary material for: “It’s harder for the likes of us”: racially minoritised stem cell donation as ethico-racial imperative
Source: Biosocieties. Author manuscript; Available in PMC 2021 Dec 1. (PMC8275909; doi:10.1057/s41292-021-00241-9)
Supplement: Supplementary Information [file EMS130402-supplement-Supplementary_Information.docx]

Table 1 Grey literature analysed

| **Year of Publication** | **Authoring Body** | **Title** |
| --- | --- | --- |
| 2010 | Stem Cell Strategic Forum | The Future of Unrelated Donor Stem Cell Transplantation in the UK: Part 1 Findings and Recommendations |
| 2010 | Stem Cell Strategic Form | The Future of Unrelated Donor Stem Cell Transplantation in the UK: Part 2 Annexes |
| 2012 | National BAME Transplant Alliance | A Voice for Change, A Vision for the Future: Strategic Plan 2012-13 to 2014-15 |
| 2014 | Anthony Nolan | Annual Report 2013/14 |
| 2014 | DKMS UK | Annual Report 2014 |
| 2014 | Anthony Nolan | 40 Years of Life Saving |
| 2014 | Anthony Nolan | State of the Registry 2014 |
| 2015 | Anthony Nolan | Annual Report 2014/15 |
| 2015 | DKMS UK | Annual Report 2015 |
| 2015 | Anthony Nolan | State of the Registry 2015 |
| 2015 | Stem Cell Oversight Committee | Unrelated Donor Stem Cell Transplantation in the UK: Effective Affordable Sustainable |
| 2016 | Anthony Nolan | Annual Report 2015/16 |
| 2016 | DKMS UK | Annual Report 2016 |
| 2016 | Anthony Nolan | Lifesaving Science: Anthony Nolan's Research Strategy |
| 2016 | Anthony Nolan | State of the Registry 2015 |
| 2017 | Anthony Nolan | Annual Report 2016/17 |
| 2017 | DKMS UK | Annual Report 2017 |
| 2017 | Anthony Nolan | State of the Registry 2016 |
| 2018 | Anthony Nolan | Annual Report 2017/18 |
| 2018 | DKMS UK | Annual Report 2018 |
| 2018 | Eleanor Smith, MP (Chair of review) | Ending the Silent Crisis: A Review into Black, Asian, Mixed Race and Minority Ethnic Blood, Stem Cell and Organ Donation |
| 2019 | Anthony Nolan | Annual Report 2018/19 |
| 2019 | Anthony Nolan | State of the Registry 2018/19 |

Table 2 Media data relating to campaigns

| Campaign name (date established) | Period of data collection^1^ | Tweets mentioned campaign^2^ | Facebook posts produced by campaign^3^ | YouTube videos produced by campaign (cumulative duration)^4^ | Blog posts published on campaign website | Print media articles discussing campaign^5^ | Radio and television articles discussing campaign (cumulative duration)^6^ |
| --- | --- | --- | --- | --- | --- | --- | --- |
| Match4Lara (Dec 2016) | February 2020 | 6,513 | 368 | 37 (51m) | 29 | 109 | 18 (1hr11mins) |
| 10,000 Donors (Aug 2018) | June 2020 | 915 | 73 | 16 (1hr51m) | 65 | 76 | 24 (2hrs37mins) |
| HelpVeerNow (Sept 2019) | October 2020 | 214 | 280 | 9 (1hr29m) | No blog | 22 | 6  (30mins) |
| ^1^All listed data for each campaign were collected within the specified periods. Campaigns listed here were at very different levels of maturity when analysed, with some having located a match, and others only recent having been established.  ^2^Tweets were scraped through WebDataRA (Web Science Institute 2020), which is limited to data that is visible to users on the Twitter website. It is therefore possible that some tweets were not captured via this method. Tweets captured were all individually read.  ^3^Posts published on the campaigns’ Facebook pages were scraped with WebDataRA and individually read.  ^4^Videos published on the campaigns’ YouTube pages were individually downloaded, watched and transcribed. Where videos were predominantly non-English language, they were excluded.  ^5^Print media were collected through Nexis and Google news searches using relevant search terms (campaign names and central individual’s name). Only English language news sources were analysed. Duplicates were removed, including syndicated newspaper content which was included only once where published in multiple local newspapers.  ^6^Radio and television coverage were located through URLs shared via campaigns’ social media and this was supplemented through Nexis searches and Box of Broadcast requests. | | | | | | | |
